# In-Hospital and Long-Term outcomes after Open-Heart Surgery in Turkish Octogenarians: a Single-Center Study

**DOI:** 10.21470/1678-9741-2020-0013

**Published:** 2021

**Authors:** Mehmet Aksüt, Deniz Günay, Tanıl Özer, Özge Altaş Yerlikhan, Emre Selçuk, Mehmet Kaan Kırali

**Affiliations:** 1 Cardiovascular Surgery Department, Kartal Kosuyolu Research and Education Hospital, Istanbul, Turkey.; 2 Cardiovascular Surgery Department, Medical Faculty of Bezmialem Vakif University, Istanbul, Turkey.

**Keywords:** Aged, 80 and over, Cardiac Surgical Procedures, Elective Surgical Procedures, Emergency Service, Hospital, Follow-Up Studies

## Abstract

**Objective:**

We aimed to analyze the early and long-term results of open-heart surgery in Turkish patients aged 80 years or older who were operated on at our center.

**Methods:**

All patients aged 80 years or older who underwent surgery between January 2000 and December 2013 at a high-level heart center were included in the study. The in-hospital data of study patients were obtained from the electronic database and from the hospital files. Survival data were analyzed as a long-term outcome.

**Results:**

A total of 245 patients aged 80-93 years were evaluated in the study. The patients were followed up 5.4±3.7 years after open-heart surgery. In-hospital mortality rates were 10% in elective cases and 15.1% overall. Age ≥85 years, chronic kidney disease, chronic obstructive pulmonary disease, and emergency surgery were independent predictors of in-hospital mortality. The median survival time was found to be 4.4±0.3 years for all participants. The long-term survival of patients who underwent emergency cardiac surgery was significantly lower than that of elective patients (log-rank <0.001).

**Conclusion:**

Octogenarians have satisfactory long-term outcomes after open-heart surgery when operated electively. On the other hand, patients operated under emergency conditions have worse in-hospital outcomes and long-term follow-up results.

**Table:** 

Abbreviations, acronyms & symbols	
CABG	= Coronary artery bypass graft
CKD	= Chronic kidney disease
CKD-EPI	= Chronic Kidney Disease Epidemiology Collaboration
COPD	= Chronic obstructive pulmonary disease
LVEF	= Left ventricular ejection fraction
SPSS	= Statistical Package for the Social Sciences

## INTRODUCTION

The number of people over 80 years old in Turkey has gradually increased, in line with worldwide statistics. From 60,000 in 1950, it reached up to 900,000 in 2015^[1]^. The worldwide cardiovascular disease prevalence is 40% among this age group; however, studies carried out in Turkey indicate that this ratio ranges from 13% to 23%^[2-6]^.

Studies carried out in developed countries have shown that early and long-term outcomes of open-heart surgery are successful in this high-risk patient group^[7-13]^. Nevertheless, the number of studies analyzing the outcomes of open-heart surgery in the Turkish octogenarian population is inadequate. A few studies have been conducted in small patient groups; the early and mid-term outcomes of these patients were analyzed, and worse outcomes were reported when compared to those in developed countries^[14,15]^.

According to current data from the Turkish Statistical Institute, however, the life expectancy of an 80-year-old Turkish person is 8 years on average, similar to that of a person of the same age in developed countries^[16]^. To overcome these contradictions, we aimed to analyze the long-term survival of Turkish octogenarians who have undergone open-heart surgery with a larger group of patients.

## METHODS

### Study Sample

The present study aimed to evaluate the data of all octogenarian patients who underwent open heart surgery at the study center between January 2000 and December 2013. The overall survival results of all patients were obtained from the national database. Therefore, data from 245 patients were analyzed in this study. The Institutional Ethics Committee approved the study protocol. The study followed the principles of the Declaration of Helsinki.

### Variables

Hypertension was defined as the active use of antihypertensive drugs or documentation of blood pressure >140/90 mmHg. Diabetes mellitus was defined as fasting plasma glucose levels >126 mg/dl, a glucose level >200 mg/dl in any measurement, or active use of antidiabetic drugs. Chronic kidney disease (CKD) was defined as estimated glomerular filtration rate <60 mL/min/1.73 m^2^. The estimated glomerular filtration rate was calculated for each patient according to the Chronic Kidney Disease Epidemiology Collaboration (CKD-EPI) equation. Chronic obstructive pulmonary disease (COPD) was defined as the active use of bronchodilator drugs or a forced expiratory volume in 1 second of <80%.

### Statistical Analysis

The distribution of continuous variables was evaluated using the Kolmogorov-Smirnov or Shapiro-Wilk tests. Categorical variables were presented as counts and frequencies; continuous variables as mean±standard deviation and minimum-maximum values. Chi-square test or Fisher’s exact test was used for comparison between categorical variables. Student’s t-test or Mann-Whitney U test was used to compare continuous variables. Survival assessment was analyzed by Kaplan-Meier survival analysis, and the log-rank test was used for comparisons. Independent predictors for in-hospital mortality were analysed with binary logistic regression. First, the univariate logistic regression model was established with well-defined operative risk factors. Likewise, every variable was included in the multivariate analyses and the SPSS software version 23.0 (SPSS, Chicago, IL) was used for all statistical analyses. When *P* was <0.05, the differences between the groups were considered statistically significant.

## RESULTS

A total of 245 consecutive patients who underwent cardiac surgery were assessed. The ages of the patients ranged from 80 to 93 years (median 81 years). It was found that 28 patients (11.4%) included in the study were 85 years of age or older; five patients (2%) were over 90 years old. Sixty-one patients (24.9%) had left ventricular ejection fractions (LVEFs) <50% (23 patients had LVEF <35%). Twenty-one patients (8.6%) had a history of cerebrovascular events, and five of them had a persistent minor neurological deficit. Four patients (1.6%) had previously undergone cardiac surgery. Twenty-six patients (10.6%) were operated on under emergency conditions, and two of them (0.8%) had acute aortic dissections. The baseline characteristics of the study patients and early postoperative outcomes are summarized in Table 1.

**Table 1:** Baseline characteristics and postoperative outcomes of the study population (n=245).

Preoperative data	
Age (years), mean±SD (min-max)	81.6±2.4 (80-93)
Male gender, n (%)	151 (6.6)
BMI (kg/m^2^) mean±SD (min-max)	26.7±4.5 (16-40)
Obesity, n (%)	28 (11.4)
Hypertension, n (%)	135 (55.1)
Diabetes mellitus, n (%)	75 (30.6)
COPD, n (%)	33 (13.5)
Renal insufficiency, n (%)	17 (6.9)
Cerebrovascular event, n (%)	21 (8.6)
Previous cardiac surgery, n (%)	4 (1.6)
Preoperative rhythm, n (%)	
Sinus rhythm	204 (83.3)
Atrial fibrillation/flutter	36 (14.7)
Pacemaker	5 (2)
LVEF (%), mean±SD (min-max)	52.1±10.9 (20-70)
**Postoperative data**	
First-day drainage (ml), mean±SD (min-max)	500±450 (100-1950)
Extubation time (hours), mean±SD (min-max)	14±8 (6-96)
Respiratory failure, n (%)	34 (13.9)
Massive bleeding, n (%)	17 (6.9)
Tamponade, n (%)	3 (1.2)
Sepsis, n (%)	11 (4.5)
Hemodialysis, n (%)	14 (5.7)
Rhythm disturbance, n (%)	52 (21.2)
Length of ICU stays (days), mean±SD (min-max)	3±2 (1-39)
Length of hospital stays (days), mean±SD (min-max)	8±6 (4-60)

BMI=body mass index; COPD=chronic obstructive pulmonary disease; LVEF=left ventricular ejection fraction; ICU=intensive care unit

Isolated cardiac procedures were performed on 203 patients (82.8%), whereas concomitant procedures were performed on 42 patients (17.2%). Seven patients (2.9%) had more than three concomitant procedures. Perfusion time and aortic cross-clamp time analyses were performed only for on-pump cardiac procedures (n=193), the mean aortic cross-clamp time was 70±34.3 (19-242) minutes, and the mean total perfusion time was 111.1±52.3 (38-480) minutes. Isolated on-pump coronary artery bypass graft (CABG) surgery (n=116, 47.3%) was the most frequently performed surgical procedure. Valve operations were performed on 73 patients (29.8%), and valve replacements (n=58) were performed rather than valve repair (n=14) (79.5% to 20.5%, respectively). The surgical procedures performed and the in-hospital mortality rates are summarized in Table 2.

**Table 2:** In-hospital survival outcomes (stratified by procedures).

	Total (n=245)	Elective (n=219)
Mortality, n (%)	37 (15.1)	22 (10)
Isolated procedures, n (%)	25/203 (12.3)	13 (7.1)
Off-pump CABG	4/52 (7.7)	4 (8.2)
On-pump CABG	14/116 (12.1)	6 (5.7)
AV surgeries	5/27 (18.5)	2 (8.3)
MV surgeries	1/5 (20)	1 (33)
Thoracic aortic surgeries	1/3 (33.3)	-
**Concomitant procedures, n (%)**	12/42 (28.6)	9 (25)
CABG + AV	5/23 (38.5)	4 (36.4)
CABG + MV	3/10 (30)	2 (22.2)
Double-valve surgeries	1/5 (20)	1 (20)
AV + ascending aortic surgeries	-	-
Three and more procedures	3/7 (42.9)	2 (40)
**Valve interventions, n (%)**		
Repair	4/15 (26.7%)	2/10 (20)
Replacement	14/58 (24.1%)	10/52 (19.2)

AV=aortic valve; CABG=coronary artery bypass graft; MV=mitral valve

### Subgroup Analyses

**Fig. 1:**
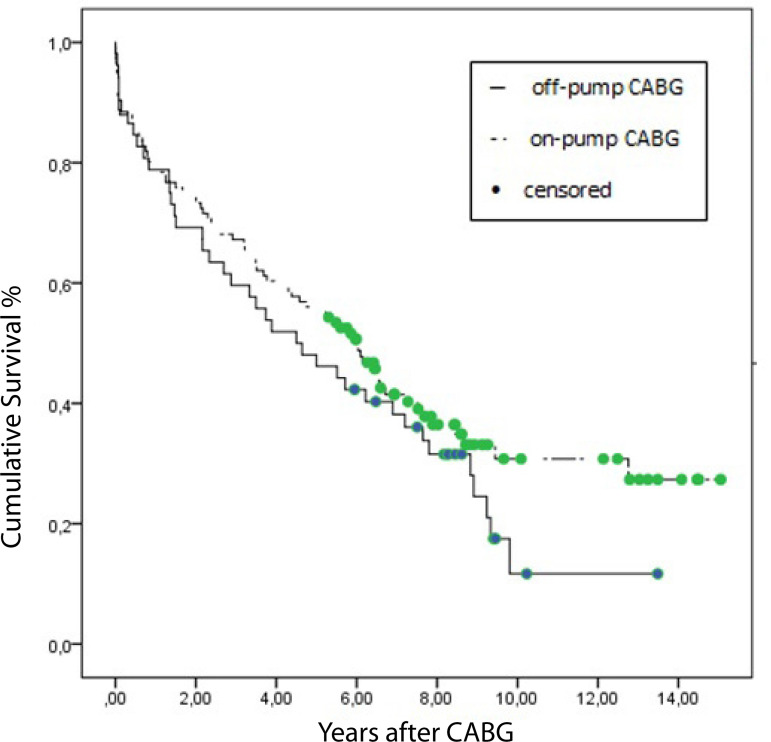
Survival curves of patients who underwent isolated coronary artery bypass surgery (7.1 years for on-pump CABG and 5.3 years for off-pump CABG, log-rank >0.05) CABG=coronary artery bypass graft.

The data of 168 patients who underwent CABG surgery are shown in Table 3. Patients were categorized as off-pump CABG and on-pump CABG. Diabetes mellitus and previous cerebrovascular event rates were significantly high in the on-pump group. In the on-pump group, more vessels were bypassed during surgery than those in the off-pump group. The mean perfusion time for patients who had isolated on-pump CABG was 97.1±30.8 minutes, and the mean ischemic time was 60.7±26.9 minutes. Eighteen out of 168 patients (10.7%) died during the in-hospital period. Analyses revealed that both techniques (on-pump CABG and off-pump CABG) had similar outcomes in the early postoperative period. The survival curves for each group are shown in Figure 1. On-pump CABG had better outcomes, but the difference was not statistically significant in the Kaplan-Meier analysis.

**Table 3:** Isolated coronary artery bypass graft surgery patients.

	On-pump CABG	Off-pump CABG	*P*-value
Patients, n (%)	116 (69)	52 (31)	-
Age (years), mean±SD	81.3±2.3	81.2±1.8	NS
Male gender, n (%)	71(61.2)	40(76.9)	NS
LITA, n (%)	85 (73.3)	36 (69.2)	NS
Graft number, mean±SD (min-max)	3±1 (1-6)	2±1 (1-4)	<0.001
Diabetes mellitus, n (%)	37 (32.5)	8 (16.7)	0.04
Hypertension, n (%)	63 (55,3)	25 (51)	NS
Renal insufficiency, n (%)	6 (5.2)	3 (5.8)	NS
COPD, n (%)	12 (10.3)	6 (11.5)	NS
Previous CVE, n (%)	15 (12.9)	1 (1.9)	0.01
Previous cardiac surgery, n (%)	1 (0.9)	0 (0)	NS
LVEF (%), mean±SD	52.6±10	48.8±11	NS
Emergency operation, n (%)	10 (8.6)	3 (5.8)	NS
First day drainage (ml), mean±SD	546.1±277	572±386	NS
Length of ICU stay (days), mean±SD	3.3±2.4	2.3±1.5	0.057
Length of hospital stay (days), mean±SD	10.2±5.5	9.2±6.5	NS
In-hospital mortality, n (%)	4 (7.7)	14 (12.1)	NS

CABG=coronary artery bypass graft; COPD=chronic obstructive pulmonary disease; CVE=cerebrovascular event; ICU=intensive care unit; LITA=left internal thoracic artery; LVEF=left ventricular ejection fraction; NS=nonsignificant

In this study, 32 patients had undergone isolated valve surgery. The mean perfusion time and the mean ischemic time were 100.9±34.6 and 63.7±27.6 minutes, respectively, in the isolated aortic valve procedures. All isolated aortic valve procedures were mechanical valve replacements. The mean perfusion and ischemic times for the mitral valve procedures, on the other hand, were 76.8±12.7 and 53.3±101 minutes, respectively. Valve repairs were performed in four (80%) patients who had undergone isolated mitral valve surgeries. The type of valve surgery technique was not related to in-hospital mortality (in-hospital mortality rate was 20% in the repair group, whereas it was 17.9% in the mechanical valve group) (*P*>0.05).

### Survival Analysis and Predictors of Mortality

**Fig. 2:**
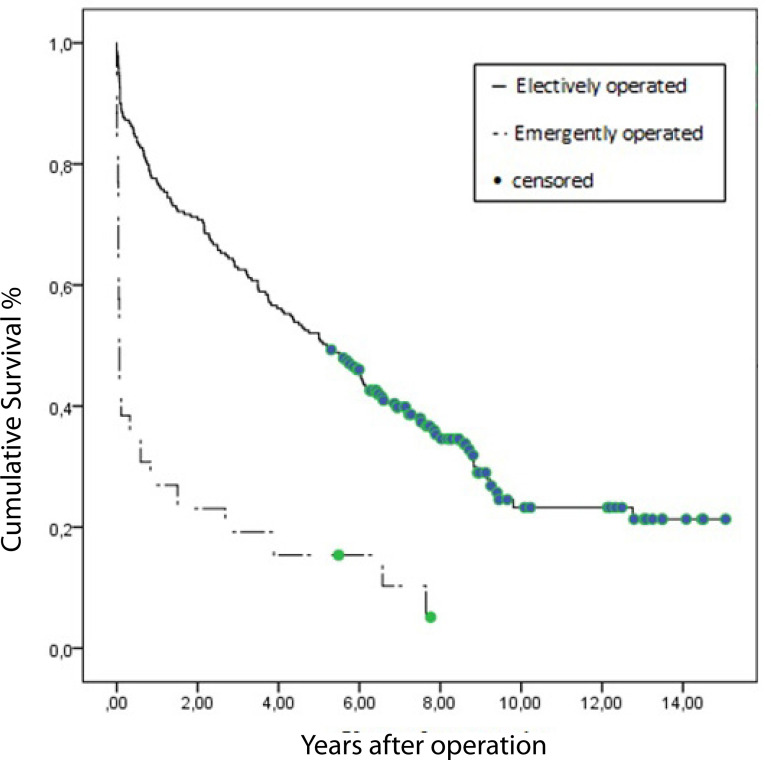
Survival curves of electively operated and emergently operated cases (5.2±0.6 years and 0.06±0.01 years, respectively, logrank <0.001).

In our study, the overall in-hospital mortality rate was 15.1%. Age ≥85 years (adjusted OR 6.08, 95% CI 2.77-11.3, *P*=0.03), chronic kidney disease (adjusted OR 2.46, 95% CI 1.17-2.75, *P*=0.003), chronic obstructive pulmonary disease (adjusted OR 2.29, 95% CI=1.92-8.06, *P*=0.02) and emergency surgery (adjusted OR 13.80, 95% CI 1.85-42.32, *P*=0.01) have a strong association with in-hospital mortality in the multivariate analysis. The in-hospital mortality rate after elective procedures was 10% and 57.7% in the patient group operated under emergency conditions. The mean follow-up was 5.4±3.7 (0.5-15) years and the median survival time was 4.4±0.3 (3.1-5.6) years for all participants (n=245). Actuarial survival rates were shown in Table 4. The median survival time, however, was 5.2±0.6 (4-6.4) years for 219 patients who had been operated on under elective conditions (Figure 2).

**Table 4:** Actuarial survival rates at 1, 5 and 10 years.

		1^st^ year	5^th^ year	10^th^ year
	**All patients**			
Total	(n=245)	176 (72%)	118 (48%)	18 (21%)
Elective cases	(n=219)	169 (77%)	114 (52%)	18 (24%)
	**Off-pump CABG**			
Total	(n=52)	41 (79%)	25 (48%)	2 (13%)
Elective cases	(n=49)	38 (78%)	24 (49%)	2 (14%)
	**On-pump CABG**			
Total	(n=116)	92 (79%)	65 (56%)	12 (31%)
Elective cases	(n=106)	90 (85%)	63 (59%)	12 (33%)
	**AV surgeries**			
Total	(n=27)	16 (59%)	11 (41%)	2 (16%)
Elective cases	(n=24)	16 (67%)	11 (46%)	2 (17%)
	**MV surgeries**			
Total	(n=5)	2 (40%)	1 (20%)	
Elective cases	(n=3)	2 (67%)	1 (33%)	

## DISCUSSION

After cardiac operations, octogenarians have worse outcomes than younger patients and in-hospital mortality rates have been found to increase up to 24%^[5,11,13,15]^. Recent studies conducted in developed countries, however, have revealed that the mortality rates of these patients have decreased to 8.1%^[10,13]^. On the other hand, in-hospital mortality rates were reported between 12.5% and 16.8% in recent studies carried out with Turkish patients^[14,15]^. In our study, the in-hospital mortality rate in electively operated cases was 10% and 7% in the isolated cardiac procedures, performed electively. These results are comparable to those of recent North American and European studies^[2,3,7-10,13,15]^.

In the present study, to avoid patient selection bias, we analyzed all octogenarian patients who had undergone cardiac surgery at our hospital. The overall in-hospital mortality rate was 15.1% in our study. We found that these patients had increased mortality rates, especially when they were operated on in emergency circumstances and concomitant procedures. Our hospital is one of the tertiary care services in the national health care system, and we cannot refuse patients at risk. We treat plenty of emergency cases from all over the country, and this situation might have affected our overall mortality rates. In our study, patients operated on in emergency conditions had an increased in-hospital mortality rate of 57.7%. Most studies concluded that patients who had undergone operations in emergency situations had in-hospital mortality rates as high as 40%^[2,5,11,12,15]^.

In subgroup analyses, the in-hospital mortality rate of the patients who had undergone isolated CABG was 10.7%. This rate decreased to 6.5% in elective cases, and was comparable to that in major studies, which defined rates between 4.1% and 13%^[7,10,13,15]^. Latest studies have shown that isolated aortic valve surgeries had in-hospital mortality rates between 5.7% and 8.5% in the octogenarian group. In our study, we found higher rates of 18.5%, but decreased to 8.3% in elective cases, which is similar to that of other studies^[2,8,12]^. Previous studies revealed that isolated mitral valve procedures had higher mortality rates than aortic valve procedures and CABG. Mortality rates from mitral valve surgeries in patients older than 80 years have been reported to be between 16.7% and 25%^[2,9]^. Similarly, in this study, this rate was found to be 20%; this was higher than the mortality rates of CABG and aortic valve operations.

In developed countries, the median survival time in octogenarians varied between 5.5 and 7.4 years after cardiac operations^[9,10]^. Although life expectancy for people aged 80 in Turkey is similar to that for people of similar age in developed countries, in our study, the mean survival time was 4.4±0.3 years for all participants, and it was lower than that obtained in other studies^[9,10,16]^. Our outcomes of in-hospital mortality rates were satisfactory in elective cases, but the median survival time of these patients was still 5.2±0.6 years. Compared to the previously published Brazilian study, which focused on isolated CABG in octogenarian population, despite the fact that the 1-year survival rates of our population was similar (80.6% and 79.4%), the 5-year survival rates of our patients were worse than that report (53.8% and 65.2%)^[17]^. A recent study has revealed that Turkish octogenarians living in urban areas have worse functional statuses than their Mexican and American counterparts^[6]^. Another community-based study suggested that Turkish octogenarians living in rural areas had functional statuses comparable to those in other developing countries^[18]^. However, due to the unbalanced distribution of cardiac centers in our country, they might have experienced difficulties in reaching satisfactory cardiac care, especially in cardiac rehabilitation and maintaining adequate international normalized ratio when using vitamin K antagonists. Besides, the inappropriate use of drugs, especially nonsteroidal anti-inflammatory drugs in chronic heart failure, was observed in the elderly Turkish population twice as high as that in developed countries^[19]^. These circumstances might have affected our long-term outcomes.

This study has several limitations due to the retrospective design of the study. Although we have done a detailed analysis of the risk profiles with defined comorbidities of the patient groups, there are probably still unmeasured variables. In particular, the insufficiency of in-hospital data for frailty assessment is one of the major deficiencies of the study. Finally, our main interest was to investigate the long-term outcomes of a challenging age group after cardiac surgery. Despite the heterogeneous study population, these results could reflect the current scenario of patients undergoing cardiac surgery.

Finally, octogenarians have satisfactory outcomes after open-heart surgery when operated electively. However, outcomes are getting worse in cases of emergency and long-term follow-up.

**Table:** 

Authors' roles & responsibilities	
MA	Substantial contributions to the conception or design of the work; or the acquisition, analysis, or interpretation of data for the work; drafting the work or revising it critically for important intellectual content; agreement to be accountable for all aspects of the work in ensuring that questions related to the accuracy or integrity of any part of the work are appropriately investigated and resolved; final approval of the version to be published
DG	Substantial contributions to the conception or design of the work; or the acquisition, analysis, or interpretation of data for the work; drafting the work or revising it critically for important intellectual content; agreement to be accountable for all aspects of the work in ensuring that questions related to the accuracy or integrity of any part of the work are appropriately investigated and resolved; final approval of the version to be published
TÖ	Substantial contributions to the conception or design of the work; or the acquisition, analysis, or interpretation of data for the work; drafting the work or revising it critically for important intellectual content; agreement to be accountable for all aspects of the work in ensuring that questions related to the accuracy or integrity of any part of the work are appropriately investigated and resolved; final approval of the version to be published
ÖAY	Substantial contributions to the conception or design of the work; or the acquisition, analysis, or interpretation of data for the work; drafting the work or revising it critically for important intellectual content; agreement to be accountable for all aspects of the work in ensuring that questions related to the accuracy or integrity of any part of the work are appropriately investigated and resolved; final approval of the version to be published
ES	Substantial contributions to the conception or design of the work; or the acquisition, analysis, or interpretation of data for the work; drafting the work or revising it critically for important intellectual content; agreement to be accountable for all aspects of the work in ensuring that questions related to the accuracy or integrity of any part of the work are appropriately investigated and resolved; final approval of the version to be published
MKK	Substantial contributions to the conception or design of the work; or the acquisition, analysis, or interpretation of data for the work; drafting the work or revising it critically for important intellectual content; agreement to be accountable for all aspects of the work in ensuring that questions related to the accuracy or integrity of any part of the work are appropriately investigated and resolved; final approval of the version to be published
